# Aluminium – Addendum: Reevaluierung des BAT-Wertes und Evaluierung einer Schwangerschaftsgruppe zum BAT-Wert: Beurteilungswerte in biologischem Material

**DOI:** 10.34865/bb742990d10_3ad

**Published:** 2025-09-29

**Authors:** Sandra Michaelsen, Wobbeke Weistenhöfer, Rüdiger Bartsch, Nadine Hund, Gerlinde Schriever-Schwemmer, Hans Drexler, Andrea Hartwig, MAK Commission

**Affiliations:** 1 Institut für Angewandte Biowissenschaften. Abteilung Lebensmittelchemie und Toxikologie. Karlsruher Institut für Technologie (KIT) Adenauerring 20a, Geb. 50.41 76131 Karlsruhe Deutschland; 2 Friedrich-Alexander-Universität Erlangen-Nürnberg. Institut und Poliklinik für Arbeits-, Sozial- und Umweltmedizin Henkestraße 9–11 91054 Erlangen Deutschland; 3 Ständige Senatskommission zur Prüfung gesundheitsschädlicher Arbeitsstoffe. Deutsche Forschungsgemeinschaft, Kennedyallee 40, 53175 Bonn, Deutschland. Weitere Informationen: Ständige Senatskommission zur Prüfung gesundheitsschädlicher Arbeitsstoffe | DFG

**Keywords:** Aluminium, Biologischer Arbeitsstoff-Toleranzwert, BAT-Wert, Entwicklungstoxizität, Entwicklungsneurotoxizität, Schwangerschaftsgruppe, aluminium, biological tolerance value, BAT value, developmental toxicity, developmental neurotoxicity, pregnancy risk group

## Abstract

The German Senate Commission for the Investigation of Health Hazards of Chemical Compounds in the Work Area (MAK Commission) re-evaluated the data for aluminium [7429-90-5] to verify the biological tolerance value (BAT value) of 50 μg aluminium/g creatinine in urine and assign it to a pregnancy risk group. Relevant studies were identified from a literature search. In the previous evaluation, neurotoxic effects were considered the most sensitive systemic endpoint of aluminium and a BAT value of 50 μg aluminium/g creatinine was derived from a no observed adverse effect level (NOAEL) of 50 μg/g creatinine for the occurrence of preclinical neurotoxic effects in humans, which was determined by standardised neuropsychological test procedures in workplace studies. As the BAT value is thus well-founded and there are no more recent data that would call this into question, the BAT value for aluminium is confirmed. Sampling time is at the end of the shift, for long-term exposures after several previous shifts. There are no reliable studies available to assess the developmental toxicity and developmental neurotoxicity of aluminium compounds in humans. There is no evidence that children are more sensitive to aluminium-induced neurotoxic effects than adults. In animal studies, aluminium concentrations in the urine of pregnant animals have not been determined and it is not known at which blood concentration developmental toxic or developmental neurotoxic effects occur in animals. There are also uncertainties regarding the transfer of animal data to humans. Therefore, a reliable risk assessment for developmental toxicity and developmental neurotoxicity is not possible and with regard to the BAT value of 50 µg aluminium/g creatinine, aluminium is assigned to Pregnancy Risk Group D.

**Table:** 

**BAT-Wert (2017)**	**50 μg Aluminium/g Kreatinin**
	Probenahmezeitpunkt: am Schichtende, bei Langzeitexposition nach mehreren vorangegangenen Schichten
Fruchtschädigende Wirkung (2024)	Gruppe D
**BAR (2018)**	**15 μg Aluminium/g Kreatinin**
	
**MAK-Wert (2024)**	**–**
**Schwerlösliche Verbindungen**	**0,05 mg Al/m^3^ A** **0,5 mg Al/m^3^ E**
Krebserzeugende Wirkung (2024)	4^a)^
Fruchtschädigende Wirkung (2024)	Gruppe D
**Lösliche Verbindungen**	**0,005 mg Al/m^3^ E für Aluminiumchlorhydrat** **0,0002 mg Al/m^3^ E für Aluminiumchlorid, Aluminiumcitrat, Aluminiumlactat, Aluminiumnitrat, Aluminiumsulfat**
Fruchtschädigende Wirkung (2024)	Gruppe C
Hautresorption (2024)	–

^a)^ Partikelüberladungseffekt in der Lunge

## Reevaluierung

Im Jahr 2017 wurden neurotoxische Effekte als empfindlichster Endpunkt des Aluminiums angesehen und aufgrund eines NOAEL von 50 μg Aluminium/g Kreatinin für das Auftreten präklinischer neurotoxischer Effekte beim Menschen, die durch standardisierte neuropsychologische Testverfahren in Arbeitsplatzstudien ermittelt wurden, ein Biologischer Arbeitsstoff-Toleranzwert (BAT-Wert) von 50 μg Aluminium/g Kreatinin abgeleitet (Klotz et al. 2018).

Im Jahr 2024 wurde aufgrund der lokalen Lungeneffekte bei Ratten (erhöhte Entzündungswerte in der BALF (bronchoalveoläre Lavageflüssigkeit), leichte Hyperzellularität und fokal-septale Kollagenablagerungen in der bronchoalveolären Region, ein erhöhtes absolutes Lungengewicht, erhöhte absolute und relative Gewichte der Lungen-assoziierten Lymphknoten und Partikel in den Alveolen und Alveolarmakrophagen) eine maximale Arbeitsplatzkonzentration **(MAK-Wert) für schwerlösliche Aluminiumverbindungen** von 0,05 mg Aluminium/m^3^ A und aufgrund der Reizwirkung an den Atemwegen im Tierversuch **MAK-Werte für lösliche Aluminiumverbindungen** von 0,005 mg Al/m^3^ E für Aluminiumchlorhydrat und 0,0002 mg Al/m^3^ E für Aluminiumchlorid, Aluminiumcitrat, Aluminiumlactat, Aluminiumnitrat und Aluminiumsulfat abgeleitet.

Da eine Exposition in Höhe des MAK-Wertes für die A-Fraktion zu einer Konzentration in biologischem Material unterhalb des BAT-Wertes führen würde, ist dieser zu reevaluieren. Zudem ist die Zuordnung des BAT-Wertes zu einer Schwangerschaftsgruppe zu prüfen.

## Toxikokinetik

Die Bioverfügbarkeit von Aluminiumverbindungen ist komplex. Sie ist abhängig von **Wasserlöslichkeit**, pH-Wert und Hydratisierung der einzelnen Aluminiumverbindungen; bei der Inhalation spielt auch die **Partikelgröße** eine Rolle. Auch Applikationsart und Applikationsdauer bedingen Unterschiede in der Bioverfügbarkeit und dem toxikokinetischen Verhalten von Aluminium. Bei Nager und Mensch bestehen Unterschiede hinsichtlich des Speichervermögens, des Kreatininbezugs und der Halbwertszeiten in Organen/Geweben (länger beim Menschen im Vergleich zur Ratte, siehe physiologisch basiertes pharmakokinetisches (PBPK)-Modell für eine einmalige orale Aufnahme; Hethey et al. 2021). PBPK-Modelle für längere Applikationsdauern liegen nicht vor. Zudem ist die erhebliche Kontaminationsgefahr in der Präanalytik hervorzuheben.

Die **orale Bioverfügbarkeit** von Aluminium ist sehr gering und beträgt beim Menschen nach Aufnahme über die Nahrung etwa 0,1 %, kann aber um den Faktor 10 variieren (EFSA 2008). Die **inhalative Bioverfügbarkeit für lösliche Aluminiumverbindungen **liegt bei 5 % (Daten von aluminiumexponierten Beschäftigen am Arbeitsplatz; Pierre et al. 1995). Für** schwerlösliche Aluminiumverbindungen **liegt die inhalative Bioverfügbarkeit niedriger bei etwa 2 % (Priest 2004).

Hohe Aluminiumgehalte finden sich besonders im Skelett. Während aus den meisten Geweben Aluminium vergleichsweise schnell freigesetzt und über die Nieren ausgeschieden wird, verläuft die Elimination aus den Knochen mit einer Halbwertszeit von mehreren Jahren sehr langsam. Unter chronischer Exposition kommt es daher zu einer** Anreicherung im Knochen** (EFSA 2008; Hellström et al. 2005).

Am Arbeitsplatz wird bezüglich der Auswahl des Probenahmezeitpunkts von einer** Akkumulation des leicht verfügbaren Aluminiums über die Arbeitswoche** hinweg ausgegangen.

Angaben zur Hintergrundbelastung der Allgemeinbevölkerung weisen **bei Serum- oder Plasmaspiegeln starke Schwankungen** auf. Laut Umweltbundesamt ist der Referenzbereich für Aluminium im Serum < 5 μg/l (HBM-Kommission 1998).

## Epidemiologische Studien 

### Effekte auf das respiratorische System

Studien zu respiratorischen Effekten nach wiederholter Exposition gegen Aluminium am Arbeitsplatz wurden in Greim (2007) ausführlich beschrieben und in Hartwig und MAK Commission (2025 a) zusammengefasst dargestellt, wobei die Ergebnisse von nach 2005 publizierten Studien ergänzt wurden.

Keine der aufgeführten Studien, die Effekte auf das respiratorische System zeigen, ist ausreichend aussagekräftig, um einen Grenzwert im biologischen Material für Lungenveränderungen ableiten zu können. Die Studie von Letzel et al. (2006) zeigte für die Jahre 1999 bis 2003 mit niedrigen gemessenen Luftkonzentrationen im Bereich von 0,47–0,76 mg aluminium(oxid)haltigem Schweißrauch/m^3^ und Medianen von 62,45–135,8 µg Aluminium/g Kreatinin im Urin und 8,7–15,56 µg Aluminium/l im Plasma (jeweils Mittelwert Vor-/Nachschicht) Lungeneffekte, die allerdings nicht allein auf Aluminium, sondern wahrscheinlich auf eine Koexposition mit Ozon oder eine Beeinflussung durch einen hohen Anteil an Rauchern oder ehemaligen Rauchern im Studienkollektiv zurückzuführen sind.

In der Studie von Hałatek et al. (2006) mit 50 Schmelzern mit Expositionen gegen Al_2_O_3_ von 0,32 ± 0,18 mg/m^3^ und im Mittel 43,7 (± 23,7) µg Aluminium/l Urin, 42 Kontrollpersonen sowie 16 weiteren gegen Aluminium exponierten Beschäftigten waren die Lungenfunktionsparameter der Schmelzer im Vergleich zu den Kontrollpersonen nicht statistisch signifikant verändert. Die Gruppe mit der höchsten Aluminiumexposition in der Luft zeigte kein statistisch signifikant erniedrigtes Keulenzell-Protein (CC16) als Zeichen chronischer Exposition. Das CC16 ist ein kleines, entzündungshemmendes Protein, das fast ausschließlich von den Keulenzellen des terminalen Bronchialepithels sezerniert wird. Zudem wird CC16 auch von anderen Faktoren wie dem Rauchen, der Umgebungstemperatur, der Tageszeit oder Infektionen beeinflusst.

Kraus et al. (2006) untersuchten 62 Arbeiter in zwei Aluminiumpulverproduktionsbetrieben und fanden mittels hochauflösender Computertomographie (HRCT) einen signifikanten Zusammenhang zwischen der Ausbildung einer Aluminose und Aluminiumkonzentrationen im Urin ab 200 µg/g Kreatinin.

### Effekte auf das zentrale Nervensystem 

Das Auftreten präklinischer neurotoxischer Effekte wurde als empfindlichster Endpunkt für die Ableitung des BAT-Wertes angesehen. Diese Effekte wurden durch standardisierte neuropsychologische Testverfahren in Arbeitsplatzstudien erfasst, die ausführlich bei Klotz et al. (2018) beschrieben wurden. Bei Hartwig und MAK Commission (2025 a) wurden Arbeitsplatzstudien ab dem Jahr 2017, weitere relevante Studien sowie Metaanalysen, Fallberichte und Patienten- und Umweltstudien dargestellt. Am Arbeitsplatz treten vor allem Expositionen gegen schwerlösliche Aluminiumverbindungen auf. In den vorliegenden Studien zeigte sich, dass die Konzentration von Aluminium im Urin nicht mit der Luftkonzentration korrelierte (Kiesswetter et al. 2007). Für lösliche Aluminiumverbindungen liegen keine epidemiologischen Studien vor (Hartwig und MAK Commission 2025 b).

Neuere Arbeitsplatzstudien aus verschiedenen Regionen Chinas zeigen sehr hohe Aluminiumgehalte im Plasma bzw. Serum sowohl bei gegen Aluminium Exponierten als auch bei nicht exponierten Kontrollpersonen (Meng et al. 2019 a, b; Shang et al. 2020, 2021). Dies deutet auf eine hohe Hintergrundbelastung mit Aluminium in den untersuchten Regionen Chinas oder eine Kontamination der Proben hin. Die Aluminiumblutspiegel in den Studien überschritten den Referenzwert der deutschen Allgemeinbevölkerung (< 5 µg/l Serum) deutlich und lagen teils im Bereich, in dem neuropsychologische Leistungsabnahmen und neurotoxische Effekte (> 13 µg/l Plasma) beobachtet wurden (Klotz et al. 2018).

An 392 männlichen Elektrolysearbeitern in China wurde der Zusammenhang zwischen Plasma-Aluminium und neurotoxischen Effekten mittels Regressionsanalyse untersucht (Zhang et al. 2022). Es wurden anhand der Aluminiumplasmaspiegel vier Gruppen mit je 98 Beschäftigten gebildet (Gruppe 1: < 18,08 µg/l Plasma (12 ± 8 Expositionsjahre), Gruppe 2: 18,08–28,2 µg/l (14 ± 10 Expositionsjahre), Gruppe 3: 28,2–40,88 µg/l (16 ± 10 Expositionsjahre) und Gruppe 4: > 40,88 µg/l (19 ± 7 Expositionsjahre)). Das Durchschnittsalter der Beschäftigten betrug 40 ± 7,4 Jahre, und sie arbeiteten im Mittel 15,1 ± 8,8 Jahre für die Firma. Die Aluminium-Staub-Konzentration wurde mit 1,07–2,13 mg/m^3^ angegeben (vermutlich E-Fraktion). In einem Fragebogen wurden Daten zum Alter, Bildungsgrad, Familienstand, Arbeitsjahre, Lebenswandel (Raucherstatus, Trinkgewohnheiten) sowie persönliche und familiäre Krankheitsgeschichten erfasst, kognitive Funktionen untersucht und der Blutdruck bestimmt. Probanden mit höheren Aluminiumkonzentrationen im Plasma schnitten im Vergleich zur Gruppe mit den niedrigsten Konzentrationen schlechter in neuropsychologischen Tests ab. Dies zeigte sich durch eine negative Korrelation zwischen Aluminiumkonzentration und den Ergebnissen im Mini-Mental-Status-Test (MMSE) (Trendtest p < 0,05) und Wortflüssigkeitstest (VFT) (Trendtest p < 0,05). Die Fuld-Objekt-Gedächtnis-Evaluation (FOME) zeigte zwar einen Trend in die gleiche Richtung, erreichte jedoch keine statistische Signifikanz. Im Gegensatz dazu war die durchschnittliche Reaktionszeit bei Probanden mit höheren Aluminiumkonzentrationen tendenziell schneller (positive Korrelation, Trendtest p < 0,05). Adjustiert wurde nach Alter, Bildungsgrad, Familienstand, Raucherstatus, Alkoholkonsum, Arbeitsjahre und Body-Mass-Index. Nur für MMSE und VFT war eine Konzentrations-Wirkungs-Beziehung bezüglich der Aluminiumplasmakonzentration modellierbar. Weiterhin hatte Gruppe 4 (> 40,88 µg Aluminium/l Plasma) im Vergleich zu Gruppe 1 (< 18,08 µg Aluminium/l Plasma) nach Adjustierung für die oben genannten Störfaktoren erhöhte Risiken für Hypertonie (Prävalenz-Ratio (PR) = 2,75; 95-%-KI: 1,24–6,09), erhöhten systolischen (PR = 2,6; 95-%-KI: 1,1–6,1) und diastolischen Blutdruck (PR = 3,36; 95-%-KI: 1,29–8,79). Die Modellierung zeigte, dass Hypertonie sowie systolischer und diastolischer Blutdruck die aluminiuminduzierte Abnahme des MMSE-Scores beeinflussen. Hypertonie hatte den stärksten Effekt (16,3 %), gefolgt von systolischem Blutdruck (14,2 %) und diastolischem Blutdruck (11,2 %). Hypertonie und diastolischer Blutdruck beeinflussten ebenfalls die aluminiuminduzierte Abnahme des VFT-Scores, mit einem jeweiligen Effekt von 9,4 % und 10,7 %. Die in dieser Studie beschriebenen Aluminiumkonzentrationen im Plasma liegen bereits in einem Bereich, in dem neurotoxische Effekte zu erwarten sind.

Die ebenfalls seit der letzten Begründung publizierten Arbeitsplatzstudien von Deschamps et al. (2009, 2018) wurden in Hartwig und MAK Commission (2025 b) ausführlich dargestellt und sind aufgrund methodischer Schwächen nicht belastbar.

Weitere Arbeitsplatzstudien können ebenfalls nicht zur Bewertung der neurotoxischen Wirkung herangezogen werden, da eine Exposition gegen weitere Metalle vorliegt (Mohammed et al. 2020; Shang et al. 2021) oder die Studien nur in chinesischer Sprache verfügbar sind (Gao et al. 2021; Li et al. 2021; Qiu et al. 2016).

Hałatek et al. (2005, 2008) untersuchten 50 Arbeiter in einer Aluminiumschmelzerei, die im Mittel Konzentrationen von 43,6 µg Aluminium/l Urin aufwiesen (95-%-KI: 37,5–50,2) und 42 Kontrollpersonen auf Veränderungen neurophysiologischer Parameter. Die Studien wurden nicht zur Bewertung herangezogen, da die verwendeten Parameter nicht dem Standard entsprechen.

Arbeitsplatzuntersuchungen von Beschäftigten der Aluminiumindustrie ohne Aluminiummessungen in der Luft aber mit Konzentrationen in Plasma und Serum finden sich in Tabelle 1.

**Tab. 1:** Neurotoxizitätsuntersuchungen an Beschäftigten der Aluminiumindustrie ohne Aluminiummessungen in der Luft

**Kollektiv,^a)^** **Land**	**Al-Konzentration Serum/Plasma** **[µg/l]**	**Ergebnis**	**Durchführung**	**Literatur**
**Querschnittstudien**				
Aluminiumverarbeitung, 831 ♂ Al-Exponierte gesamt Alter: Gruppe 1: 207 (40,25 ± 8,32 Jahre) Gruppe 2: 208 (41,52 ± 7,87 Jahre) Gruppe 3: 208 (40,46 ± 7,96 Jahre) Gruppe 4: 208 (39,66 ± 7,33 Jahre) Expositionsjahre: Gruppe 1: 16,37 ± 9,87 Gruppe 2: 17,36 ± 9,61 Gruppe 3: 15,84 ± 10,31 Gruppe 4: 14,85 ± 8,63, China Arbeiter trugen Schutzkleidung, Masken, Schutzbrille, Mundschutz und Handschuhe; Al-Konzentration im Trinkwasser < Richtwert der WHO (200 µg/l)	Plasma: MW: 15,26 Gruppe 1: < 8,28 Gruppe 2: 8,28–15,26 Gruppe 3: 15,26–27,02 Gruppe 4: > 27,02	MW, Vergleich Gruppe 1 und 4: stat. sig. im Trend-Test** und in partieller Korrelationsanalyse*: Gesamt-CDT-Score ↓, visuospatiale u. exekutive Funktionen ↓, adjustiert nach Alter, Bildung, Einkommen, Familienstand, Arbeitsplatz, Trink/Rauchgewohnheiten	**Fragebogen:** Alter, Geschlecht, Bildungsgrad und Einkommen, Familienstand, Lebenswandel (Raucherstatus, Trinkgewohnheiten), Berufsgeschichte, Krankheitsgeschichte und Medikamenteneinnahme, Familienanamnese **Ausschlusskriterien:** kognitive Beeinträchtigungen hervorrufende Erkrankungen, bezüglich Demenz positive Familienanamnese, regelmäßige Medikamenteneinnahme (mit Al oder ZNS betreffend), Seh-/Hör-Probleme **Tests zur kognitiven Funktion:** MMSE, CDT, multi-domain-cognition	Wang et al. 2020
Aluminiumverarbeitung, 172 ♂ Al-Exponierte (Alter: 40,89 ± 5,77 Jahre), Expositionsjahre: 6,64 ± 6,37 245 Kontrollen (Alter: 41,63 ± 5,38 Jahre), China Arbeiter trugen Arbeitskleidung, Masken und Handschuhe; Al-Konzentration im Trinkwasser < nationaler Standard (< 200 µg/l)	Plasma (Median (P25–P75)): Al-Exponierte: 21,18 (11,84–40,54) n = 55: < 14,9 n = 117: > 14,9 Kontrollen: 10,46 (5,32–19,24) n = 153: < 14,9 n = 92: > 14,9	Testergebnisse (MW ± SD) Al-Exponierte ↓**: MMSE: 27,93 ± 1,91 CDT: 2,70 ± 1,03 DS: 10,97 ± 1,96 FOME: 23,60 ± 3,12 Kontrollen: MMSE: 28,62 ± 1,25 CDT: 3,16 ± 0,86 DS: 12,24 ± 2,15 FOME: 25,80 ± 2,84 multivariate Regressionsanalyse: Al-Plasma-Spiegel ↑: erhöhtes Risiko für Beeinträchtigungen in CDT* (OR: 1,79; 95-%-KI: 1,13–2,84), für Gedächtnis u. Lernen** (OR: 1,88; 95-%-KI: 1,2–2,9), für visuell-räumliche u. exekutive Funktionsstörungen (OR: 2,02; 95-%-KI: 1,11–3,66); adjustiert nach Alter, Bildungsgrad, Einkommen, Rauch- und Trinkgewohnheiten	**Ausschlusskriterien:** kognitive Beeinträchtigungen hervorrufende Erkrankungen, in Bezug auf Demenz positive Familienanamnese, regelmäßige Medikamenteneinnahme (mit Al oder ZNS betreffend), Seh-/Hör-Probleme, fehlende demographische Daten u. Blutproben **Tests zur kognitiven Funktion:** MMSE, CDT, DS, FOME	Meng et al. 2019 b
Aluminiumverarbeitung, 853 ♂ Beschäftigte, davon 53 Fälle mit MCI (Alter: 45,04 ± 6,15 Jahre), Expositionsjahre: < 1 (n = 34) > 1 (n = 19) 212 Kontrollen (Alter: 44,71 ± 6,11 Jahre), China Arbeiter trugen Arbeitskleidung, Masken und Handschuhe; Al-Konzentration im Trinkwasser < nationaler Standard (< 200 µg/l)	Plasma (Median (P25–P75)): Al-Exponierte mit MCI: 18,17 (10,39–34,96) n = 18: < 13,13 n = 35: > 13,13 Kontrollen: 12,02 (6,35–20,86) n = 114: < 13,13 n = 98: > 13,13	Multivariate logistische Regressionsanalyse: Al-Plasma-Spiegel ↑: Risiko kognitiver Beeinträchtigung ↑ (AOR*: 2,24; 95-%-KI: 1,17–4,26), adjustiert für Bildung, Rauch- und Trinkgewohnheiten Raucherstatus u. Alkoholkonsum: keine Assoziation mit kognitiver Beeinträchtigung	**Ausschlusskriterien:** kognitive Beeinträchtigungen hervorrufende Erkrankungen, in Bezug auf Demenz positive Familienanamnese, regelmäßige Medikamenteneinnahme (mit Al oder ZNS betreffend), Benutzung Al-haltiger Kochutensilien, Seh-/Hör-Probleme, fehlende demographische Daten und Blutproben **Tests zur kognitiven Funktion:** MMSE, CDT (Werte nicht dargestellt)	Meng et al. 2019 a
Aluminiumproduktion, 576 ♂ Al-Exponierte, davon 85 Personen mit MCI (MMSE < 24 Punkte, CDT < 3) (Alter: 39,86 ± 10,09 Jahre), 85 Kontrollen (Alter: 40,15 ± 9,46 Jahre) Arbeitsjahre: Al-Exponierte mit MCI: 16,91 ± 9,02 Kontrollen: 16,53 ± 8,56, Asien (k. w. A.)	Serum (MW ± SD): Al-Exponierte mit MCI: 85,18 ± 23,68 (↑**) Kontrollen: 33,13 ± 14,11	Al-Exponierte mit MCI: MMSE ↓*, CDT ↓*, adjustiert nach potentiellen Störfaktoren (k. w. A.) *PI3K/Akt/mTOR1*-Genexpression ↓*	**Fragebogen:** demographische Daten (z. B. Alter, Geschlecht), Daten zum Arbeitsplatz (z. B. Arbeitsjahre, Schutzkleidung), Krankheitsgeschichte, Lebensgewohnheiten (z. B. Alkoholkonsum, Rauchen) **Ausschlusskriterien:** Alter > 60 Jahre, Bildung geringer als Primarschule, Beschäftigungsdauer in Al-Produktion < 10 Jahre, Diagnose einer mentalen oder neurodegenerativen Erkrankung, neurodegenerative Erkrankungen in Familienanamnese, Einnahme von Psychopharmaka > 1 Monat, Einnahme von Antazidum und/oder Al-enthaltenden Nahrungsmitteln oder Verwendung von Aluminiumkochgeschirr > 1 Monat, starke Lärmbelastung, Aphasie oder Schwerhörigkeit, extrem unkoordinierter oder kranker Eindruck **Tests zur kognitiven Funktion:** MMSE, CDT	Shang et al. 2020
Aluminiumelektrolyse, 187 ♂ Al-Exponierte, davon 49 ohne MCI (Alter: 41 Jahre (35,5–45,5)) 138 mit MCI (Alter: 42,5 Jahre (35–48)), Asien	Plasma (Median (IQR)): Gruppe ohne MCI: 55,86 (38,70–77,01) Gruppe mit MCI: 72,79 (42,51–102,65)	total MoCA score: Gruppe ohne MCI: ≥ 26 Gruppe mit MCI: < 26	**Fragebogen und Ausschlusskriterien:** siehe Shang et al. 2020 **Tests zur kognitiven Funktion:** MoCA	Shang et al. 2021
Aluminiumverarbeitung, 1660 ♂ Al-Exponierte Alter [Jahre]: jüngere Beschäftigte (< 40 Jahre, k. A. zur Anzahl) Gruppe 1: 32,35 ± 4,89 Gruppe 2: 32,77 ± 5,18 Gruppe 3: 30,41 ± 5,30 Gruppe 4: 32,99 ± 4,43 ältere Beschäftigte (≥ 40 Jahre, k. A. zur Anzahl) Gruppe 1: 45,41 ± 3,70 Gruppe 2: 45,54 ± 3,72 Gruppe 3: 45,43 ± 3,70 Gruppe 4: 44,83 ± 3,40 Arbeitsjahre: jüngere Beschäftigte Gruppe 1: 8,94 ± 6,27 Gruppe 2: 10,77 ± 6,52 Gruppe 3: 8,34 ± 5,69 Gruppe 4: 15,28 ± 5,64 ältere Beschäftigte Gruppe 1: 21,10 ± 7,32 Gruppe 2: 21,16 ± 8,47 Gruppe 3: 22,22 ± 7,00 Gruppe 4: 22,47 ± 8,53, China Arbeiter trugen Schutzkleidung, Masken und Schutzbrillen	Plasma: je n = 415 Gruppe 1: < 15 Gruppe 2: 15,0–34,52 Gruppe 3: 34,52–42,25 Gruppe 4: > 42,25	Multiple lineare Regressionsanalyse/Trend-Test: stat. sig.* negative Korrelation von DST, DSBT mit Plasma-Al-Konzentration bei allen Beschäftigten sowie bei Älteren und Jüngeren, adjustiert nach Alter, Bildungsstand, Familienstand, Alkoholkonsum, Rauchgewohnheiten und Arbeitsjahre; logistische Regressionsanalyse: OR der Gruppe 4: jüngere Beschäftigte DSBT: 15,31 (95-%-KI: 4,18–56,06)**, DST: 3,27 (95-%-KI: 1,62–6,62)** ältere Beschäftigte DSBT: 7,64 (95-%-KI: 3,85–15,19)**, DST: 1,7 (95-%-KI: 1,06–2,71)*	**Fragebogen:** Alter, Bildungsstand, Familienstand, Alkoholkonsum, Rauchgewohnheiten, weitere sozioökonomische und Lebensstil-Faktoren, **Ausschlusskriterien:** fehlende Blutproben oder Messungen der Aluminiumkonzentration im Plasma, Beschäftigungszeit < 1 Jahr, regelmäßige Einnahme aluminiumhaltiger Magenmedikamente, nicht kooperativ, psychische oder neurologische Erkrankungen **Tests zur kognitiven Funktion:** MMSE, CDT, DST, FOME, VFT, SRT, ATIME, FAS, SLO	Xu et al. 2021
Aluminiumschmelzung, 66 ehemals Al-Exponierte (Alter: 62,03 ± 7,09 Jahre), 70 Kontrollen (Alter: 60,77 ± 7,95 Jahre) Arbeitsjahre: Al-Exponierte: 30,18 ± 7,23 Kontrollen: 31,54 ± 5,98 Alkoholkonsum (mehr als 3mal/Woche in den letzten 6 Monaten): Al-Exponierte: 72,7 % Kontrollen: 61,4 %; China	Serum (MW ± SEM): ehemals Al-Exponierte: 25,18 ± 2,65 ↑** Kontrollen: 9,97 ± 2,83	Kognitive Funktion: MMSE (adjustiert nach Alter und Bildungsgrad) Exponierte: 26,13 ± 2,57 ↓**, Kontrolle (27,89 ± 1,91) MCI-Fälle: Exponierte: 12 (18,2 %); Kontrollen: 4 (5,7 %) MCI-Fälle gegen nicht-MCI: stat. sig.* ↑: tau5, p-tau181, p-tau231, p-tau396 Al-Exponierte gegen Kontrollen: stat. sig.* ↑ p-tau231, p-tau181	**Gruppiert nach:** Alter, Bildungsgrad, sozioökonomischer Status, Lebensstil, Gesundheit. **Fragebogen:** Alter, Bildungsgrad, Lebensstil (Raucherstatus, Trinkgewohnheiten), Berufsgeschichte, eigene und familiäre Krankheitsgeschichte **Ausschlusskriterien:** regelmäßige Medikamenteneinnahme (z. B. Antazida), Kopftrauma, Nierenschäden, Seh-/Hör-Probleme, psychiatrische, somatische, neurologische Erkrankung **Tests zur kognitiven Funktion:** MMSE **Untersuchung peripherer Blutlymphozyten:** Proteinexpression von t-tau (tau5), p-tau396, p-tau262, p-tau-231, p-tau181	Lu et al. 2014
**Longitudinalstudien**				
Aluminiumverarbeitung, 276 Al-Exponierte (♂) (Alter: 37,9 ± 7,8 Jahre) gesamt, Gruppe 1: n = 91 (38,0 ± 8,2 Jahre) Gruppe 2: n = 93 (38,8 ± 7,5 Jahre) Gruppe 3: n = 92 (36,8 ± 7,6 Jahre) Arbeitsjahre: Gesamt: 11,3 ± 8,2 Gruppe 1: 12,0 ± 8,7 Gruppe 2: 12,3 ± 8,5 Gruppe 3: 9,5 ± 7,2, China	Plasma (Bereich): Median (P25–P75): Gesamt: 27,69 (12,47–46,01), Gruppe 1 (< 17,6): 7,82 (4,51–12,39) Gruppe 2 (17,6–37,3): 27,58 (22,50–32,92) Gruppe 3 (> 37,3): 63,01 (46,01–93,19)	Generalisierte lineare Regression 2014: keine stat. sign. Assoziation zwischen Plasma-Al-Konzentration und kognitiven Leistungen 2016–2014: Plasma-Al-Konzentration negativ assoziiert mit MMSE2016–2014*, DSBT2016–2014*, FOME2016–2014**, VFT2016–2014* multivariate logistische Regression: ↑ Plasma-Al-Konz. erhöht Risiko einer FOME-Score-Abnahme stat. sig. **, adjustiert nach Alter, Bildungsgrad, Familienstand, Trink/Rauchgewohnheiten, Dienstjahre, Arbeitsplatz	**Ausschlusskriterien:** bekannte mentale oder neurologische Beeinträchtigungen, Medikamenteneinnahme (Antazida), fehlendes Follow-up, weniger als 2 Jahre am Arbeitsplatz beschäftigt, nicht kooperativ **Fragebogen:** Alter, Geschlecht, Bildungsgrad, Familienstand, Lebensgewohnheiten (Raucherstatus, Trinkgewohnheiten), Krankheitsgeschichte **Tests zur kognitiven Funktion** (2014 und Follow up 2016): MMSE, CDT, ATIME, FOME, DS, DSFT, DSBT, VFT, FAS, SLO	Lu et al. 2021

* p < 0,05;

** p < 0,01; Al: Aluminium; AOR: adjustiertes Odds-Ratio; ATIME: average reaction time; CDT: Clock drawing test; CFT: Category fluency repetition; DS: Digit span test; DSBT: Digit span backward test; DSFT: Digit span forward test; FAS: Fastest reaction time; FOME: Fuld Object-Memory Evaluation; MCI: Mild cognitive impairment; MMSE: Mini-mental state examination; MoCA: The Montreal Cognitive Assessment; MW: Mittelwert; P25: 25. Perzentil; P75: 75. Perzentil; SD: Standardabweichung; SEM: Standardfehler; SLO: Slowest reaction time; SRT: Simple reaction time; stat. sig.: statistisch signifikant; VFT: Verbal fluency Test

^a)^ Al-Serum-/Plasmaspiegel in Kontroll- bzw. Vergleichsgruppen aller Studien vergleichsweise hoch (Referenzwert Allgemeinbevölkerung < 5 µg/l (HBM-Kommission 1998))

## Reevaluierung des BAT-Wertes

Es gibt kaum Informationen über durch Aluminiumstaub verursachte Lungenerkrankungen, die meisten davon stammen aus der Aluminiumpulverindustrie. Von den aufgeführten Studien, die Effekte auf das respiratorische System zeigen, ist keine aussagekräftig genug, um daraus einen Grenzwert für Lungenveränderungen ableiten zu können. Ein statistisch signifikanter Zusammenhang zwischen der Ausbildung einer Aluminose und Aluminium wurde bei Konzentrationen im Urin ab 200 µg Aluminium/g Kreatinin beobachtet (Kraus et al. 2006).

Die MAK-Werte für schwerlösliche und lösliche Aluminiumverbindungen wurden für die lokalen Effekte in der Lunge abgeleitet (Hartwig und MAK Commission 2025 a, b). Der BAT-Wert für Aluminium orientiert sich an der Neurotoxizität, die aus einer systemischen inneren Belastung resultiert. Neurotoxische Effekte wurden als empfindlichster systemischer Endpunkt des Aluminiums angesehen und aufgrund eines NOAEL von 50 μg/g Kreatinin für das Auftreten präklinischer neurotoxischer Effekte beim Menschen, der durch standardisierte neuropsychologische Testverfahren in Arbeitsplatzstudien ermittelt wurde, ein BAT-Wert von 50 μg Aluminium/g Kreatinin abgeleitet (Klotz et al. 2018). Da der BAT-Wert somit gut begründet ist und keine neueren Daten vorliegen, die dies in Frage stellen würden und zur Grenzwertableitung herangezogen werden können,

**wird der BAT-Wert für Aluminium im Urin von 50 μg Aluminium/g Kreatinin bestätigt.**

Die Probenahme ist am Schichtende, bei Langzeitexposition nach mehreren vorangegangenen Schichten.

## Interpretation

Aluminiumverbindungen weisen sehr unterschiedliche Löslichkeiten auf. Es wurden daher MAK-Werte für schwerlösliche Aluminiumverbindungen von 0,05 mg Al/m^3^ für die A-Fraktion (0,5 mg Al/m^3^ für die E-Fraktion) und für lösliche Aluminiumverbindungen von 0,005 mg Al/m^3^ E für Aluminiumchlorhydrat und 0,0002 mg/m^3^ E für Aluminiumchlorid, Aluminiumcitrat, Aluminiumlactat, Aluminiumnitrat und Aluminiumsulfat abgeleitet. Die Messung der Luftkonzentrationen gestaltet sich dabei häufig problematisch. Zudem liegt oft ein sehr heterogenes Gemisch von Aluminiumverbindungen vor (z. B. beim Aluminiumschweißen). Beim Biomonitoring wird der bioverfügbare Anteil von leicht- und schwerlöslichen Aluminiumverbindungen erfasst. Bei einer inhalativen Exposition gegen Aluminiumverbindungen ist daher zur sicheren Bewertung der beruflichen Belastung nicht nur die Luftkonzentration zu messen, sondern auch ein Biomonitoring durchzuführen. Andererseits entbindet das Einhalten des BAT-Wertes den Arbeitgeber nicht von der Einhaltung des Luftgrenzwertes, insbesondere bei den löslichen Aluminiumverbindungen.

Dieser BAT-Wert schützt nicht vor Reizerscheinungen oder lokalen Effekten löslicher Aluminiumverbindungen.

In der präanalytischen Phase ist eine Kontamination der Proben durch die Verwendung von geeigneten Urinbechern, die erst direkt vor der Probenahme geöffnet werden, und das Versenden der Urinprobe in diesem Becher ohne nochmaliges Umfüllen weitgehend zu verhindern.

## Fruchtschädigende Wirkung

Für Stoffe, deren MAK- oder BAT-Wert von einer neurotoxischen Wirkung abgeleitet wurde, muss die Entwicklungsneurotoxizität bewertet werden. Der BAT-Wert für Aluminium wurde an der Neurotoxizität abgeleitet.

## Epidemiologische Studien

Es liegen einige umweltepidemiologische Studien zur Aluminiumbelastung schwangerer Frauen vor (Tabelle 2; siehe auch Hartwig und MAK Commission 2025 a, b).

**Tab. 2:** Umweltepidemiologische Studien zu Aluminiumkonzentrationen im Urin schwangerer Frauen

**Land**	**Kollektiv (Alter: MW ± SD; Bereich),** **Zeitpunkt der Urinprobe**	**Aluminium im Urin** **[µg/l]**	**Analytik**	**Bemerkungen**	**Literatur**
British Columbia, Kanada	31 ♀ (21–41 Jahre), ca. 18,5. SSW, Pilotstudie	GM: 15,3 P10: 5,15 P95: 355,0	ICP-MS; NWG: k. A.; Urinproben an 5 aufeinanderfolgenden Tagen	Al-Messungen von 29 ♀	Caron-Beaudoin et al. 2019
Houston, Texas, USA	131 ♀ (mind. 18 Jahre), sozioökonomisch benachteiligte schwangere Frauen	GM: 23,3 MW ± SD: 45,1 ± 120,7 Bereich: 2,12–1346,64	ICP-MS; NWG: 0,66 μg/l; Spontanurin	Al-Messungen von 126 ♀	Han et al. 2020
West Australien	173 ♀ (19–44 Jahre), ca. 2 Wo. vor Entbindung, Nichtraucherinnen	MW: 13,1 Median: 9,1 Bereich: < 5,0–78,7 P5: < 5,0 P95: 42,7	ICP-MS; NWG: 5,0 μg/l		Callan et al. 2013
Israel	beduinisch-arabische Herkunft, > NWG: 15 ♀ (30,8 ± 7,1; 20–41 Jahre), < NWG: 43 ♀ (27,6 ± 6,1; 20–42 Jahre), bei Ankunft zur Entbindung im Krankenhaus	GM: 12 Bereich: 9–28,3	GF-AAS; NWG: 9 μg/l; Spontanurin	Assoziation zwischen Al u. geringerer Körpergröße der Neugeborenen	Karakis et al. 2014
Israel	beduinisch-arabische Herkunft, > NWG: 37 ♀ (30,8 ± 7,1; 20–41 Jahre), < NWG: 103 ♀ (k. A.), bei Ankunft zur Entbindung im Krankenhaus	Proben > NWG: GM: 12,2 95-%-KI: 10,9–12,6 Bereich: 7,2–28,3	GF-AAS; NWG: 9 μg/l; Spontanurin	Assoziation zwischen Al u. geringfügigen Anomalien	Karakis et al. 2015
Israel	beduinisch-arabische Herkunft, 141 ♀ (28,1 ± 6,1 Jahre), bei Ankunft zur Entbindung im Krankenhaus	GM: 6,95 95-%-KI: 4,63–10,43 Maximum: 412,43	ICP-MS; NWG: 0,01 μg/l; Spontanurin		Karakis et al. 2020
Israel	beduinisch-arabische Herkunft, 110 ♀ (28,1 ± 6,3; 18,4–41,7 Jahre), bei Ankunft zur Entbindung im Krankenhaus	GM: 6,14 95-%-KI: 3,80–9,90 Bereich: 0,01–97,27	ICP-MS; BG: 0,01 μg/l; Spontanurin	Assoziation zwischen Al u. vorzeitiger Geburt bzw. dem Auftreten von Fehlbildungen	Karakis et al. 2021
Südafrika	450 ♀ (24,8 ± 6,2; 14–49 Jahre), bei Ankunft zur Entbindung im Krankenhaus	GM: 13,1 95-%-KI: 11,97–14,35 MW ± SD: 18,1 ± 14,9 Bereich: 2,21–106,3	ICP-MS; NWG: 1,71 μg/l	Al-Messungen von 318 ♀	Röllin et al. 2018
Französisch-Guyana	Geophagie-Gruppe (langzeitige Ernährung von Ton; Hb ≤ 85 g/l): 98 ♀ (26 ± 7,3 Jahre), Kontrollgruppe (Hb > 105 g/l): 85 ♀ (27 ± 6,4 Jahre), ca. 2. Trimester	Geophagie-Gruppe: MW ± SD: 92,8 ± 251,2; Kontrollgruppe: MW ± SD: 12,1 ± 23	ICP-MS; NWG: k. A.	Pemba (Tonprodukt zur Ernährung) als Al-Quelle	Lambert et al. 2010
Mexiko-City, Mexiko	188 ♀ (k. A. zum Alter), 3. Trimester	AM: 37,6; GM: 25,3 Median: 24,6 IQR: 14,6; 43,9 Maximum: 333	ICP-MS; BG: 8,6 μg/l		Lewis et al. 2018
Wuhan, China	746 ♀ (28,6 ± 3,3 Jahre) im 1. Trimester (13. SSW), davon 745 ♀ im 2. Trimester (24. SSW) 599 ♀ im 3. Trimester (35. SSW)	1. Trimester: GM: 23,6 95-%-KI: 21,4–26,0, 2. Trimester: GM: 20,4 95-%-KI: 18,0–23,2, 3. Trimester: GM: 28,4 95-%-KI: 25,0–32,7	ICP-MS; NWG: 1,06 μg/l; Spontanurin		Liu et al. 2019

AAS: Atomabsorptionsspektroskopie; AM: arithmetischer Mittelwert; BG: Bestimmungsgrenze; GF: Graphitrohr; GM: geometrischer Mittelwert; ICP-MS: induktiv gekoppelte Plasma-Massenspektrometrie; IQR: Interquartilbereich; k. A.: keine Angabe; KI: Konfidenzintervall; MW: Mittelwert; NWG: Nachweisgrenze; P5: 5. Perzentil; P10: 10. Perzentil; P95: 95. Perzentil; SD: Standardabweichung; SSW: Schwangerschaftswoche

Es liegen keine belastbaren Untersuchungen zur Bewertung der fertilitätsschädigenden und entwicklungstoxischen Wirkung von Aluminiumverbindungen beim Menschen vor.

Bei Kindern und Erwachsenen kam es durch die Verwendung von aluminiumkontaminierten Dialysaten oder aluminiumhaltigen Phosphatbindern (bei Urämie) zu einer erheblichen Aluminiumbelastung. Typischerweise lagen die Aluminiumkonzentrationen im Plasma bei 100 bis 200 µg/l; bei schweren Fällen bei über 500 µg/l. Bei einzelnen Patienten wurde dadurch ein aluminiuminduziertes neurotoxisches Syndrom, was auch als „Dialyse-Demenz“ bezeichnet wird, ausgelöst (ATSDR 2008). Aus den vorliegenden Studien lässt sich nicht ableiten, ob Kinder empfindlicher als Erwachsene reagieren. Diese Studien sind nicht zur Bewertung der Toxizität von Aluminium am Arbeitsplatz geeignet.

Aluminiumkonzentrationen im Urin trächtiger Tiere wurden nicht bestimmt. Die Daten zur Aluminiumkonzentration im Blut trächtiger Tiere ermöglichen keine Aussage, bei welcher Blutkonzentration entwicklungs-/entwicklungsneurotoxische Effekte auftreten.

Der plazentare Übergang von Aluminium vom Muttertier zum Fetus wurde für Ratten und Mäuse gezeigt. Die Menge des Aluminiums, die beim Fetus ankommt, ist abhängig von der Konzentration im maternalen Blut. Eine quantitative Abschätzung ist aufgrund fehlender Daten nach Verabreichung über die gesamte Trächtigkeit hinweg nicht möglich.

Zwischen Nager und Mensch bestehen große Unterschiede bei der Toxikokinetik von Aluminiumverbindungen (siehe Hethey et al. 2021), so dass hinsichtlich einer toxikokinetischen Umrechnung große Unsicherheiten bestehen.

**Für den BAT-Wert von 50 µg Aluminium/g Kreatinin ist eine belastbare Risikoabschätzung zur Entwicklungs- und Entwicklungsneurotoxizität nicht möglich.**

Die Argumente für die Zuordnung des BAT-Wertes von Aluminium zur neuen Schwangerschaftsgruppe B (Verdacht) wurden in der Kommission kritisch diskutiert. Ein Verdacht auf Schwangerschaftsgruppe B ließ sich aus den vorliegenden Daten nicht begründen.

Folgende Argumente sprechen für eine Zuordnung des BAT-Wertes für Aluminium zur Schwangerschaftsgruppe D:

1. Variabilität der Aluminiumbelastung durch Lebensstil, ubiquitäres Vorkommen von Aluminium
2. Variabilität der Aluminiumkonzentrationen im Urin von Schwangeren, keine Aussage zur Entwicklungsneurotoxizität möglich
3. keine Hinweise, dass Kinder empfindlicher gegen aluminiuminduzierte neurotoxische Effekte reagieren als Erwachsene
4. Aluminiummenge, die beim Fetus ankommt, ist vermutlich abhängig von der Konzentration im maternalen Blut (EFSA 2008). Bei einer Halbwertszeit von 5 Stunden ist keine bedeutende Akkumulation im Blut zu erwarten.
5. keine Bestimmung von Aluminiumkonzentrationen im Urin trächtiger Tiere; Blutkonzentration unbekannt, bei der entwicklungstoxische/entwicklungsneurotoxische Effekte beim Tier auftreten
6. Unsicherheiten bei der Übertragung von Tierdaten auf den Menschen

Daher wird Aluminium bei einem **BAT-Wert von 50 μg Aluminium/g Kreatinin der Schwangerschaftsgruppe D** zugeordnet.
